# Externally Applied Electromagnetic Fields and Hyperthermia Irreversibly Damage Cancer Cells

**DOI:** 10.3390/cancers15133413

**Published:** 2023-06-29

**Authors:** Elena Obrador, Ali Jihad-Jebbar, Rosario Salvador-Palmer, Rafael López-Blanch, María Oriol-Caballo, María Paz Moreno-Murciano, Enrique A. Navarro, Rosa Cibrian, José M. Estrela

**Affiliations:** 1 Department of Physiology, Faculty of Medicine and Odontology, University of Valencia, 46010 Valencia, Spain; elena.obrador@uv.es (E.O.); ajijeb@alumni.uv.es (A.J.-J.); rosario.salvador@uv.es (R.S.-P.); loblanch@alumni.uv.es (R.L.-B.); maria.oriolc@gmail.com (M.O.-C.); rosa.m.cibrian@uv.es (R.C.); 2 Scientia BioTech, 46002 Valencia, Spain; paz.moreno72@gmail.com (M.P.M.-M.); enrique.navarro@uv.es (E.A.N.); 3 Department of Computer Sciences, Higher Technical School of Engineering, 46100 Burjassot, Spain; 4 IRTIC Institute, University of Valencia, 46980 Paterna, Spain; 5 Department of Physiology, Faculty of Pharmacy, University of Valencia, 46100 Burjassot, Spain

**Keywords:** non-ionizing radiations, electromagnetic fields, hyperthermia, cancer therapy, cancer cell death

## Abstract

**Simple Summary:**

Moderate loco-regional hyperthermia (40–45 °C) is a therapeutic modality that can improve the effects of chemotherapy and radiotherapy and, in addition, improve our immune response against cancer. However, the effects of these combinations on many different cancers are modest. The use of much higher temperatures (>60 °C, thermal ablation) can cause severe damage to healthy tissues. In fact, our results show that cancer cells show extraordinary resistance to moderate hyperthermia. However, we found that the combination of hyperthermia (not higher than 52 °C) with low-strength electromagnetic fields acts synergistically, causing irreversible damage to different cancer cells. Moreover, these externally applied energies can be combined with chemotherapy and/or targeted therapies to achieve complete cancer eradication. In vivo, the energy causing focal hyperthermia can be distributed in multiple beams that can be concentrated in the tumor, thus avoiding damaging the healthy tissues that it passes through.

**Abstract:**

At present, the applications and efficacy of non-ionizing radiations (NIR) in oncotherapy are limited. In terms of potential combinations, the use of biocompatible magnetic nanoparticles as heat mediators has been extensively investigated. Nevertheless, developing more efficient heat nanomediators that may exhibit high specific absorption rates is still an unsolved problem. Our aim was to investigate if externally applied magnetic fields and a heat-inducing NIR affect tumor cell viability. To this end, under in vitro conditions, different human cancer cells (A2058 melanoma, AsPC1 pancreas carcinoma, MDA-MB-231 breast carcinoma) were treated with the combination of electromagnetic fields (EMFs, using solenoids) and hyperthermia (HT, using a thermostated bath). The effect of NIR was also studied in combination with standard chemotherapy and targeted therapy. An experimental device combining EMFs and high-intensity focused ultrasounds (HIFU)-induced HT was tested in vivo. EMFs (25 µT, 4 h) or HT (52 °C, 40 min) showed a limited effect on cancer cell viability in vitro. However, their combination decreased viability to approximately 16%, 50%, and 21% of control values in A2058, AsPC1, and MDA-MB-231 cells, respectively. Increased lysosomal permeability, release of cathepsins into the cytosol, and mitochondria-dependent activation of cell death are the underlying mechanisms. Cancer cells could be completely eliminated by combining EMFs, HT, and standard chemotherapy or EMFs, HT, and anti-Hsp70-targeted therapy. As a proof of concept, in vivo experiments performed in AsPC1 xenografts showed that a combination of EMFs, HIFU-induced HT, standard chemotherapy, and a lysosomal permeabilizer induces a complete cancer regression.

## 1. Introduction

The biological effects of non-ionizing electromagnetic fields (EMFs) have been extensively investigated for decades [1]. Based on the guidelines of the International Commission on non-Ionizing Radiation Protection, “Radiofrequency Electromagnetic Fields” (RF EMFs) is the term used to describe the part of the electromagnetic spectrum comprising the frequency range from 100 kHz to 300 GHz (https://www.icnirp.org (accessed on 18 October 2022)). Electric fields result from differences in voltage, whereas magnetic fields result from the flow of electric current. Tumor-treating fields (TTFs) are a type of oncotherapy that uses alternating electric fields of intermediate frequency (∼100–500 kHz) and low intensity (1–3 V/cm) to disrupt cell division and promote inhibition of cancer growth [2]. The use of this range of intermediate frequency is based on evidence showing that low-frequency electric fields (<1 kHz) progressively disrupt cell membrane polarization, whereas high-frequency fields (>1000 kHz) cause heating-induced damages due to the vibration of the charged/polar cell molecules [3,4]. The established technique for applying the TTFs requires placing electrodes on the body surface around the focus of tumor growth so that a potential difference can be generated across growing cancer [5] (https://www.novocure.com (accessed on 7 April 2022)). Charged molecules, if subjected to an electric or an EMF, are affected by the direction and intensity of the energy flow. However, there are key differences in the way electric and magnetic fields interact with charged molecules, i.e., (but not limited to) (a) the electric field lines are in the direction of the voltage gradient and do not form a loop, whereas the magnetic field lines are around the currents forming a closed loop; (b) the electric field is inversely proportional to the voltage gradient, whereas the intensity of the magnetic field depends on the number of field lines produces by the magnet and the current enclosed in its loop; (c) the electric field lines are measured in two dimensions, whereas the magnetic field lines are measured in three dimensions (https://www.niehs.nih.gov (accessed on 23 January 2023)). At present, potential applications of non-thermal EMFs on cancer therapy have not been implemented yet [6].

Molecular vibration increases as temperature increases and hyperthermia (HT) can damage and kill cancer cells. However, in practice, HT-based applications in oncotherapy still face strong limitations [7]. These limitations include (a) HT can cause damage to healthy tissues surrounding the tumor if the temperature is not precisely controlled (e.g., burns, blistering, and necrosis); (b) patients may experience pain, discomfort, or a burning sensation during the HT treatment; (c) HT may cause skin reactions such as redness, swelling, or rash in the treated area; (d) limited penetration depending on the source and type of energy applied; (e) the response to HT can vary among individuals and tumor types (some tumors may be more sensitive to heat, while others may be less responsive); (f) ensuring precise targeting of the tumor area while avoiding damage to surrounding healthy tissues is challenging. Thus, advanced imaging techniques and treatment planning are necessary to improve targeting accuracy. Based on the guidelines of the US National Cancer Institute, HT is a type of treatment in which body tissue is heated to as high as 45 °C to help damage and kill cancer cells with little or no harm to normal tissue (http://www.cancer.gov (accessed on 1 August 2021)). To this end, suggested techniques include probes that generate energy from microwaves, radio waves, lasers, ultrasounds, perfusion of heating fluids, or heating of an entire body in a heated chamber or hot water (http://www.cancer.gov (accessed on 10 October 2022)). All these approaches have potential side effects and limited efficacy [8].

Although electromagnetic and heat energies may affect many different cell functions (e.g., [9]), their association is still underdeveloped as a potential oncotherapy. Magnetic nanoparticles-based HT (used as nano-heaters activated by an external magnetic field) has been investigated. However, the development of efficient heat nanomediators with high specific absorption rate values is essential to overcome some key restrictions [i.e., non-specificity, bioavailability, and toxicity] [10]. Up to now, these restrictions have precluded nanoparticles-based HT from reaching clinical practice.

In the present report, we demonstrate that low-range EMFs and HT (without the use of nanomediators) irreversibly damage different cancer cells. To this end, our experimental setup had to meet two principles: (a) the highest magnetic field strength that does not cause a measurable temperature rise in a thermostated environment at 37 °C (the internal temperature in mammals), and (b) HT should be applied during specific periods of time (which can be achieved in vivo and locally by means, e.g., of high-intensity focused ultrasounds, HIFU). Our results suggest that this approach may help overcome the limitations of HT in oncotherapy.

## 2. Materials and Methods

### 2.1. Cell Culture

Human A2058 (melanoma), AsPC1 (pancreatic adenocarcinoma), and MDA-MB-231 (a hormone-independent breast adenocarcinoma) cells were from the American Type Culture Collection (Manassas, VA, USA). Human AsPC1/Luciferase Stable Cells were obtained from GenTarget Inc. (San Diego, CA, USA). Cells were grown in DMEM (Invitrogen, Waltham, MA, USA), pH 7.4, supplemented with 10% heat-inactivated fetal calf serum (FCS) (Biochrom AG, Berlin, Germany), 100 U/mL penicillin, and 100 µg/mL streptomycin. Cells were plated (20,000 cells/cm^2^) and cultured at 37 °C in a humidified atmosphere with 5% CO_2_. Cells were harvested by incubation for 5 min with 0.05% (*w*/*v*) trypsin (Merck, Darmstadt, Germany) in phosphate-buffered saline, pH 7.4, containing 0.3 mM EDTA, followed by the addition of 10% FCS to inactivate the trypsin. Cell number and viability were determined using a BioRad (Hercules, CA, USA) TC20 Automated Cell Counter. Cell integrity was also confirmed by measuring leakage of lactate dehydrogenase activity. Cells were allowed to attach for 24 h before any treatment addition.

### 2.2. Experimental Setup for the Combined Treatment with EMFs and HT In Vitro

The source of EMFs was generated with a Promax-GFG-8216 generator (GW Instek, Taipei, Taiwan), adjusted for an output signal of f (frequency) = 100–500 KHz, with an amplitude of 2 V. This signal can be visualized with an oscilloscope to check for accuracy of amplitude and f of the sinusoidal signal. The output of the generator was connected with a BCN connector to a coaxial cable of 50 Ω of characteristic impedance. The end of it was soldered to a WE-760308102142 coil from Würth Electronik (Rot am See, Germany). According to the manufacturer’s information, the coil has an inductance L = 5.8 µH and a DC resistance (or ohmic resistance of a conductor) of 0.01 Ω. The calculated skin resistance at, e.g., 100 KHz is 0.014 Ω. The coil consists of two superimposed windings, with two parallel copper wires forming a 5-winding flat spiral.

The current can be easily calculated based on the generator output voltage and the coil impedance obtained from the manufacturer, and the magnetic field can be calculated with this current and its geometrical distribution. The WE-760308102142 coil is mathematically modeled as two sets of 10 concentric turns of increasing radius, one set above the other. The outer diameter of the coils was 48.85 mm, and the copper wire had a diameter of 1.5 mm. The magnetic induction B-field (Tesla) along the axis is calculated as follows:

$$B=\frac{\mu_{0}I}{2}\left[\sum_{k=1}^{n}\frac{a_{k}{}^{2}}{{\left(a_{k}{}^{2}+z_{1}^{2}\right)}^{3/2}}+\sum_{k=0}^{n}\frac{a_{k}{}^{2}}{{\left(a_{k}{}^{2}+z_{2}^{2}\right)}^{3/2}}\right]$$

where

$$I=\frac{V}{\sqrt{R^{2}+{\left(L\omega -1/C\omega \right)}^{2}}}$$

μ_0_. Permeability of the free space (vacuum) 1.257 × 10^−6^ (Henry/m).
z_1_. Height above the first layer of coil turns (mm).
z_2_ = z_1_ + 0.75 mm.
*n* = 10. Number of turns of each layer of the coil.
a_k_. Radius of each loop (mm).
R. Resistance (Ohm).
V. Output voltage of the generator (Volt).
𝜔 (angular frequency) = 2πf, being f the frequency (Hertz).
L. Inductance of the coil (Henry).
C. Parasitic capacitance of the coil (Farad).

The mathematical equation calculates the magnetic induction B-field (Tesla) along the axis of the coil at 3 mm from its surface (approx. 25 µT).

The culture flasks (T25) were placed just above the coil so that the distance between it and the base of the flask was approx. 3 mm. The coil and flask were wrapped in a plastic bag, which was immersed in a thermostated water bath. The temperature of the culture medium was controlled by means of a thermal probe (IKA ETS-D5 temperature controller, Merck, Darmstadt, Germany) placed through the screw cap of the flask. Under these experimental conditions, the thermostated water flowing around the culture flask maintained the temperature of the culture medium within the value determined for each experimental condition.

### 2.3. Flow Cytometry and Cell Death Analysis

Cell cycle, viability, and death were analyzed with a BD FACSVERSE (Becton Dickinson, Franklin Lakes, NJ, USA). Cell death was measured using propidium iodide and Annexin V-FITC (Thermo Fisher Scientific, Waltham, MA, USA) following the procedure recommended by the manufacturer.

Apoptotic and necrotic cell death was also distinguished using fluorescence microscopy [11]. To this end, cells were incubated for 3 min with Hoechst 33342 (10 mM; which stains all nuclei) and propidium iodide (10 mM; which stains nuclei of cells with a disrupted plasma membrane) and then analyzed using a Diaphot 300 fluorescence microscope (Nikon, Tokyo, Japan) with excitation at 360 nm. Nuclei of viable, necrotic, and apoptotic cells were detected as blue round nuclei, pink round nuclei, and fragmented blue or pink nuclei, respectively. About 1500 cells were counted each time. The DNA strand breaks in apoptotic cells were assayed using a direct TUNEL labeling assay (Merck) and fluorescence microscopy following the manufacturer’s methodology.

### 2.4. Cytochrome c, Apoptosis-Inducing Factor, and Heat-Shock Proteins

Cancer cells were washed twice with phosphate-buffered saline, and the pellet was suspended in ice-cold homogenization buffer (2 × 10^6^ cells per ml of buffer: 20 mM HEPES pH 7.5, 250 mM sucrose, 1 mM MgCl_2_, 10 mM KCl, 1 mM EDTA, 1 mM EGTA, 1 mM dithiothreitol, 0.1 mM phenylmethylsulfonyl fluoride, and 10 mg leupeptin, aprotinin, and pepstatin A/mL). The cells were homogenized with a Dounce homogenizer. After centrifugation at 2500× *g* for 5 min at 4 °C, the supernatants were centrifuged at 100,000× *g* for 30 min at 4 °C. The resulting supernatant was used as the soluble cytosolic fraction (SCF). Proteins were quantified [12], separated by SDS-PAGE, transferred to nitrocellulose membranes, and probed with anti-cytochrome c (Cyt C) (ab110325, abcam, Cambridge, UK), anti-AIF (sc-55519, Santa Cruz Biotechnology, Santa Cruz, CA, USA), anti-heat-shock protein (Hsp) 70 (ab194360, abcam) and anti-Hsp110 (ab108625, abcam) monoclonal antibodies. Blots were developed using horseradish peroxidase-conjugated secondary antibody and enhanced chemiluminescence (ECL system; GE HealthCare Life Sciences, Malborough, MA, USA). Protein bands were quantitated using a Bio-Rad Gel Doc Go Imaging System.

### 2.5. Mitochondrial Membrane Potential

Quantitative determination of the mitochondrial membrane potential (MMP) was performed by the uptake of the radiolabeled lipophilic cation methyl triphenylphosphonium (TPMP), which enables small changes in the potential to be determined [13]. Briefly, cancer cells (2 × 10^6^) were incubated at 37 °C for 60 min in 1 mL DMEM, supplemented as mentioned above but including 1 mM TPMP, 250 nCi [^3^H]TPMP (Amersham, Bucks, UK), and 1 mM sodium tetraphenylboron. After incubation, the cells were pelleted by centrifugation (1000× *g* for 5 min), 100 mL of the supernatant was removed, the pellet was resuspended in 100 mL 10% Triton X-100, and the radioactivity (disintegrations/min) was measured using a Tri-Carb Liquid Scintillation Counter from Perkin-Elmer (Waltham, MA, USA). Non-specific TPMP binding was corrected as previously described [13]. Energization-dependent TPMP uptake was expressed as an accumulation ratio in units of [(TPMP/mg protein)/(TPMP/mL supernatant)] [14].

### 2.6. Oxygen Consumption

O_2_ concentration and consumption in isolated cancer cells were measured using an oxygraph of OROBOROS Instruments (Innsbruck, Austria) and as previously described [15].

### 2.7. H_2_O_2_ and O_2_^−^

Quantitative measurement of H_2_O_2_ and O_2_^−^ generation followed the previously described methodology [15].

### 2.8. Cancer Cell Compartmentation

Cytosolic (cyt) and mitochondrial (mt) compartments were rapidly separated, as previously reported in detail for cancer cells [16], using digitonin and centrifugation through a layer of silicon oil.

### 2.9. ATP

ATP levels were measured fluorometrically following standard enzymic methods [17].

### 2.10. Glutathione

Glutathione (GSH) was determined by LC/MS, as previously reported [18]. Cell processing was performed according to the published methodology, where rapid N-ethylmaleimide derivatization was used to prevent GSH auto-oxidation [19].

### 2.11. Caspase 3

This activity was measured using a highly sensitive colorimetric substrate, N-acetyl-Asp-Glu-Val-Asp p-nitroanilide (Ac-DEVD-pNA), following the manufacturer’s instructions (CalBiochem, La Jolla, CA, USA). Briefly, cancer cells were lysed in lysis buffer [50 mM HEPES (pH 7.4), 100 mM NaCl, 0.1% (*v*/*v*) CHAPS, 1 mM dithiothreitol and 0.1 mM EDTA] on ice for 10 min, then centrifuged at 10,000× *g* for 10 min at 4 °C. Equal volumes of the supernatants were added to equal volumes of assay buffer [50 mM HEPES (pH 7.4), 100 mM NaCl, 0.1% (*v*/*v*) CHAPS, 10 mM dithiothreitol, 0.1 mM EDTA, and 10% glycerol] and incubated at 37 °C for 10 min. Then, freshly prepared Ac-DEVD-pNA (200 mM) was added to the mixture, and A405 was monitored every 20 min for 3 h at room temperature. Cultures without cell lysates were used as controls. Enzyme activity was calculated using the manufacturer’s formulae as pmol/min.

Z-DEVD-FMK (Z-Asp-Glu-Val-Asp-fluoromethylketone; CalBiochem), dissolved in DMSO and added in a 0.2% volume to give the concentration indicated in Section 3, was used as an irreversible caspase 3 inhibitor.

### 2.12. Lysosomal Membrane Integrity

We used LysoTracker™ Deep Red (Thermo Fisher Scientific), a deep red-fluorescent dye for labeling and tracking acidic organelles in live cells. Fluorescence microscopy was run at 577/590 nm (excitation/emission). All the procedure was performed following the manufacturer’s recommendation.

### 2.13. Cathepsin Activities

Cancer cell lines were seeded in T25 flasks and, 24 h later, were treated as indicated in Section 3. After the removal of the medium, an extraction buffer containing different concentrations of digitonin (Merck) was used to separate cytosolic and total cathepsins. When necessary, the concentration of digitonin was optimized for different cell types. Cells were incubated with ice-cold lysis buffer (CelLytic^TM^ MT Mammalian Tissue Lysis/Extraction Reagent) containing a protease and phosphatase inhibitors cocktail (Merck) for 15 min at 4 °C on a rocking platform. Cysteine (Cys) and aspartate (Asp) cathepsin activities were measured using the fluorescent substrates zFR-AFC (AFC = 7-Amino-4-trifluoromethylcoumarin) (excitation at 405 nm; emission at 510 nm) and MCA-GKPILFFRLK(Dnp)-DR-NH2 [MCA = (7-methoxycoumarin-4-yl)acetyl; Dnp = dinitrophenyl] (excitation at 320 nm; emission at 420 nm) (Enzo Biochem, New York, NY, USA), respectively. Pepstatin A (5 mg/mL) and Leupeptin (50 mg/mL) (Merck) were used to inhibit the activity of aspartyl peptidases and serine-cysteine proteases, respectively.

### 2.14. Gene Silencing

Human Hsp70-specific small hairpin RNA (shRNA) was obtained and transfected following the methodology described by Zhu et al. for hepatocellular carcinoma HepG2 cells [20].

### 2.15. Tumor Xenografts

AsPC1 cells, cultured and harvested as explained above, were washed and resuspended in DMEM and inoculated subcutaneously in the lateral part of the body (5 × 10^6^ cells/nu-nu mouse, female, 12 weeks old, Charles River Laboratories). Mice were fed a standard diet (Letica, Rochester Hills, MI, USA). Tumor growth was measured every 2 days using calipers. Tumor volume was calculated in mm^3^ based on the following formula, volume = 0.5a × b^2^, where a and b are the long and short diameters, respectively. Bioluminescence detection of cancer cell activity was performed by injecting (i.p.) IVIS brite D-Luciferin Potassium (150 mg/kg, Perkin-Elmer, Waltham, MA, USA) into AsPC1/Luciferase Stable Cells -bearing mice. Bioluminescence was detected using an IVIS Spectrum In Vivo Imaging System (Perkin-Elmer).

### 2.16. Experimental Setup for In Vivo Treatment

Our experimental setup was based on a technique previously described by Park et al. [21]. Mice were anesthetized with isoflurane, placed and fixed on a methacrylate platform, and their body immersed (up to the neck) in a degassed water bath (24 L, 40 × 30 × 20 cm) thermostated at 37 °C. A solenoid (similar to that used in vitro, see above) was placed close to the tumor to ensure that it received approx. 25 µT. The HIFU was composed of two spherical piezoelectric elements with 1.0 MHz resonance frequency, 500 W of acoustic potency (rms), and a diameter of 60 mm (Scientia BioTech, Valencia, Spain). The piezoelectric elements were placed to emit 2 ultrasonic beams that impacted the tumor, one vertically to the tumor and the other perpendicular to the previous one. This arrangement was designed in such a way that the energy dissipated was as less damaging as possible to non-cancerous tissues. In the center of one of the piezoelectric elements, an ultrasound scanner was used as a guide to monitor the image of the tumor. This system allows 3D tissue reconstruction for planning and 2D imaging for monitoring during treatment. The system was adjusted so that the two ultrasonic beams emitted by the piezoelectric elements hit approximately the center of the tumor. During treatment with non-ionizing radiations, no noticeable motion was observed in the real-time monitored ultrasound images of the tumor. Mice were treated once per day for three consecutive days. In each session, mice were subjected for 40 min to the combined effect of EMFs and the HIFU-induced HT. The total energy generated by the two HIFU transducers in the tumor was that corresponding to a potency of approximately 60 W/cm^2^. To make sure that under our experimental condition, a temperature of approximately 52 °C was reached within the tumor, in previous control experiments, a thermocouple (TE connectivity, Schaffhausen, Switzerland) was inserted in different AsPC1 tumors in vivo. Then the tumors were subjected to HIFU radiation to make sure which potency was necessary to reach the required temperature. The aim of this protocol was to maximize the anticancer effect as much as possible while taking into account the limitations of the in vivo model. Before each session in the bath, mice were pretreated × 12 h with EMFs in animal housing cages. These cages were surrounded by an attached network of copper cables that allowed the tumor-bearing mice to receive approximately 25 µT constantly. The aim of this procedure was to try to maximize the effect of the EMFs on the growing tumor. Gemcitabine (50 mg/kg) was administered 1 h before each 3-day treatment period (see Section 3).

### 2.17. Pterostilbene Levels

The analysis was performed using liquid chromatography and mass spectrometry (LC-MS/MS) as previously described [22].

### 2.18. Evaluation of Therapy-Induced In Vivo Toxicity

This included the following parameters: animal weight, complete blood cell count, and standard blood chemistry.

### 2.19. Statistical Analysis

Data are presented as mean values ± SD for the number of different experiments. Data were analyzed by one- or two-way analysis of variance (ANOVA) or unpaired *t*-tests where appropriate (SPSS Statistics 29 for Windows; SPSS Inc., Chicago, IL, USA). The homogeneity of the variances was analyzed by the Levene test. The null hypothesis was accepted for all the values of the tests in which the F value was nonsignificant at *p* > 0.05. The data for which the F value was significant was examined by Tukey’s test at *p* < 0.05.

## 3. Results

### 3.1. EMFs and HT Decrease Cancer Cell Viability

As explained in the introduction and based on the experimental setup (see under Section 2), exposure to EMFs was not associated with an increase in the cell culture temperature above 37 °C. On the other hand, the protocol to generate HT under in vitro conditions was designed thinking of its potential application in vivo. An eventual in vivo approach to increase the tumor temperature should be a) rapid and specifically focused on the tumor and b) on a scale of temperature that should cause limited damage to normal tissues near the tumor. To this end, we subjected the cancer cells to a range of temperatures from 37 °C to a maximum of 52 °C. This range of temperature is easy to reach with different methodologies and (if correctly focused on the tumor) should cause limited side effects in normal peritumoral tissues. As a proof of concept, we inoculated subcutaneously, AsPC1 cancer cells into nude immunodeficient mice (*n* = 5) [22]. Two weeks after inoculation, the tumor volume reached 75–100 mm^3^, and it was heated in vivo for 20 or 40 min with an experimental HIFU device bearing a single transducer (Holosonic S.L., Valencia, Spain). We observed that (a) the internal temperature of the animal (controlled by a thermal probe placed in the rectum) remained below 38 °C, whereas (b) the temperature of the tumor (controlled by a probe placed on the peritumoral skin) could be increased up to 52 °C in less than 1 min. The spatial shape of the HIFU beam was Gaussian, providing enough accuracy in heating the tumor; therefore, only the skin surrounding the tumor showed inflammation (grade 2 after 20 min and grade 3 after 40 min of HIFU treatment). Inflammation was scored from 0 to 4 as follows: 0 (none), 1 (apparent increase in polymorphonuclear leukocytes in vessels and migration of these cells into the adjacent tissue in the vicinity of the vessels), 2 (more diffuse, but still relatively sparse inflammation), 3 (intermediate between 2 and 4) and 4 (maximum density of polymorphonuclear leukocytes). Once the HIFU energy was stopped, the tumor temperature returned to 37 °C in less than 2 min, a temperature decrease favored by the well-known physiological heat-loss mechanisms. This preliminary in vivo experiment confirmed that focused HT (even as high as 52 °C) is feasible and may have limited side effects.

**Figure 1:**
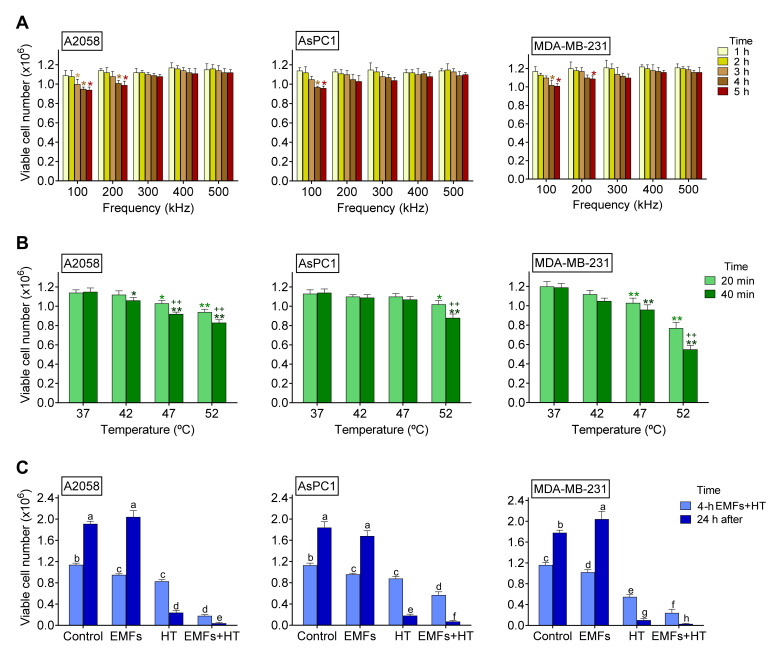
Effect of EMFs and HT on cancer cell viability. (**A**) Effect of EMFs. Cancer cells were seeded and 24 h later exposed to EMFs (100–500 kHz × 1–5 h). Control values (0 kHz) were 1.14 ± 0.03 A2058, 1.13 ± 0.04 AsPC1, and 1.20 ± 0.05 MDA-MB-231 (×10^6^) viable cells (*n* = 5 in all cases). * *p* < 0.05 comparing all conditions versus controls (0 kHz) (*n* = 5 *t*-test). (**B**) Effect of HT. Cancer cells were seeded and 24 h later exposed to HT (42–52 °C × 20–40 min). * *p* < 0.05 ** *p* < 0.01 comparing all conditions versus controls (37 °C) ^++^ *p* < 0.01 comparing 40 min versus 20 min (*n* = 5 *t* test). (**C**) Effect of EMFS and HT. Cancer cells were seeded and 24 h later exposed to EMFs (100 kHz × 4 h) and HT (52 °C × 40 min from min 120 to min 160 of the 4 h period where cells were constantly exposed to the EMFs). The surviving cells were cultured for 24 additional hours without further exposure to EMFs and HT. A two-way analysis of variance (ANOVA) was used to make comparisons among the different groups after 4 h of treatment with EMFs + HT and 24 h after. Different letters indicate differences *p* < 0.05 (*n* = 5).

As shown in Figure 1A, under in vitro conditions, EMFs (100–200 kHz × 4 h) very slightly affect the viability of three different cancer cell lines. HT up to 52 °C × 40 min caused a significant (although limited) decrease in viability (to approximately 72% in A2058, 77% in AsPC1, and 46% in MDA-MB-231 cells of control values) (Figure 1B). However, as shown in Figure 1C, the combination of EMFs and HT caused a much higher decrease in cell viability (to approx. 16%, 50%, and 21% of controls values in A2058, AsPC1, and MDA-MB-231 cells, respectively) after the 4 h protocol described in the caption of Figure 1. Importantly, in the following 24 h period, the tumor cell population did not recover, and viability further decreased to approximately 2.1% (A2058), 3.8% (AsPC1), and 1.6% (MDA-MB-231) of control values (Figure 1C); thus, indicating that the damage caused by the combination of non-ionizing radiations (NIR) is severe. Nevertheless, this dramatic decrease in cell viability could be misleading since cancer cells, under in vivo conditions, may implement mechanisms to resist the effect of NIR, adapt, survive, and grow again. Moreover, in vivo, the complex structures surrounding the tumor (stroma, vasculature, and other cells), plus paracrine and systemic factors, may favor their survival. We know that even a small % of a surviving tumor can follow (a posteriori) an explosive growth pattern. Thus, it is key to consider that the combination of EMFs and HT may not be enough and should be combined with other oncotherapies.

**Figure 2:**
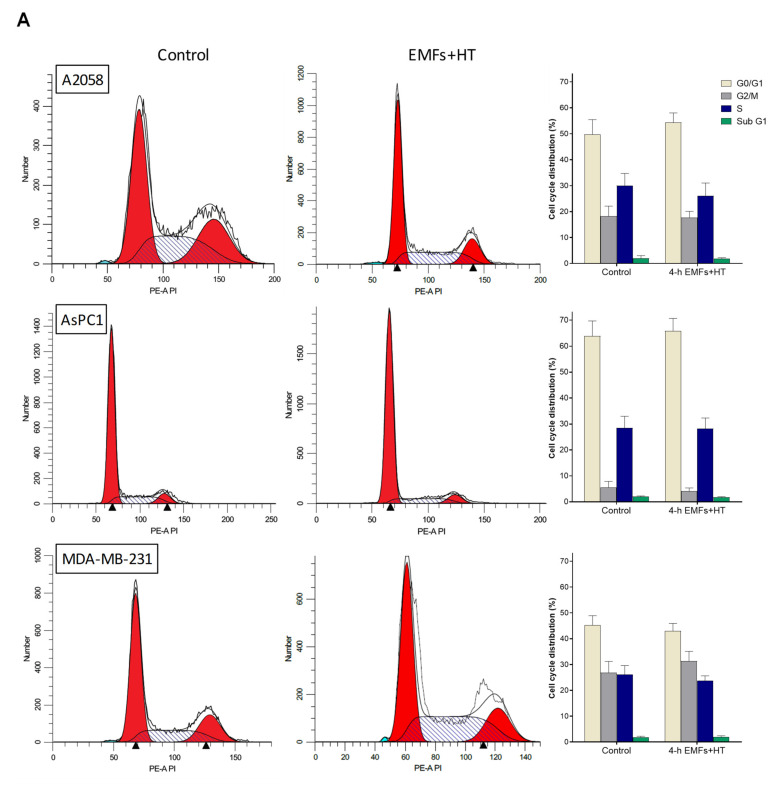

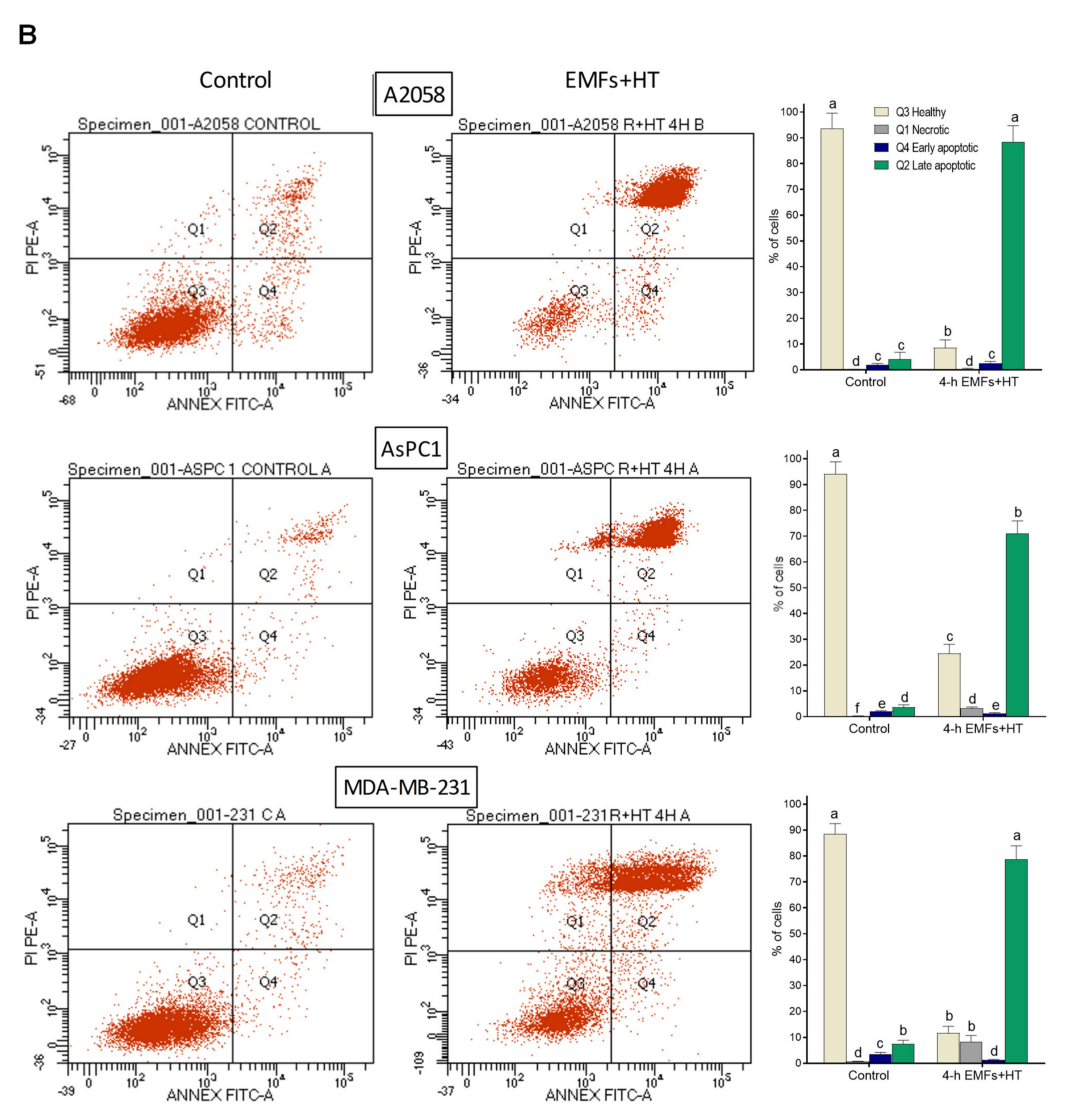
Effect of EMFs and HT on cell cycle distribution and the type of death in cancer cells. (**A**) Flow cytometry analysis of the cell cycle distribution after exposure to EMFs and HT as in Figure 1C (*n* = 5). No statistically significant differences were found when comparing treatment with EMFs + HT and controls. (**B**) Flow cytometry analysis of EMFs and HT-induced apoptosis and necrosis (treatment as in Figure 1C). A two-way analysis of variance (ANOVA) was used to make comparisons among control cells treated with EMFs + HT and the different cell subpopulations in both groups. Different letters indicate differences *p* < 0.05 (*n* = 5).

**Figure 3:**
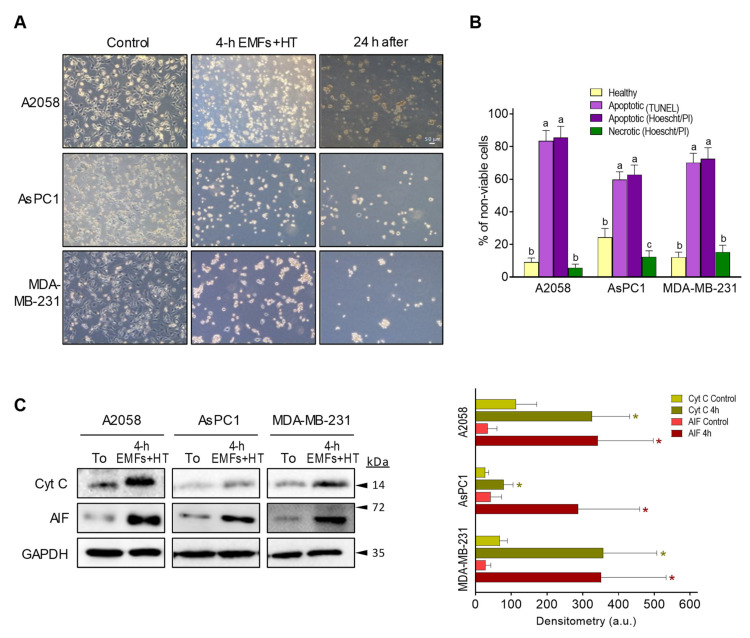
Effect of EMFs and HT on the activation of apoptotic death in cancer cells. (**A**) Inverted microscope images (magnification ×10) of cancer cells treated with EMFs and HT (as in Figure 1C) showing the drastic morphological changes associated with the loss of viability. (**B**) Cell death analysis after the 4 h protocol (Figure 1C) based on Hoechst 33342 and propidium iodide (PI) staining and the TUNEL labeling assay (see under Methods). Cell viability in control flasks was > 98% in all cases. A one-way analysis of variance (ANOVA) was used to make comparisons among cell subsets. Different letters indicate statistical differences *p* < 0.05 (*n* = 5). (**C**) Western blots for detection of cytochrome C and AIF in the cytosolic fraction (all performed right after the 4 h protocol Figure 1C). Densitometric analysis (a.u. arbitrary units) represents the mean values ± SD for 5 different experiments per cell line [* *p* < 0.01 comparing cells treated with EMFs and HT (4 h as in Figure 1C) versus untreated controls *t*-test]. The original western blot figures could be found in Figure S3.

As shown in Figure 2A, treatment with EMFs and HT (4 h protocol, as in Figure 1C) did not cause significant changes in the cell cycle distribution of the cancer cells studied. However, it is remarkable that EMFs and HT-induced loss of cell viability are mainly associated with massive apoptosis (Figure 2B). The effect of EMFs or HT assayed separately, did not change this trend, e.g., in the case of A2058 cells, the small decrease in cell viability induced by EMFs or HT (Figure 1B) is also associated with apoptosis (approximately a 71 ± 7% of non-viable cells in the case of EMFs and 87 ± 6% in the case of HT were identified as apoptotic, *n* = 5 in both cases). Inverted microscope images showed that EMFs + HT treatment causes drastic changes in the shape of cultured cancer cells (Figure 3A). Importantly, cells treated with EMFs and HT did not recover in the following 24 h (Figure 3A), suggesting that (a) the damage caused to the cancer cells is not reversible and (b) the few remaining cells could possibly be in a position of particular weakness against the cytotoxic effect of chemotherapeutic drugs. Cell death analysis (Figure 3B) and the increase in the cytosolic detection of apoptosis-inducing factor and cytochrome C (Figure 3C) further confirmed the EMFs and HT-induced activation of apoptotic cell death.

### 3.2. EMFs and HT Increase ROS Generation and the Release of Death Signals from Mitochondria

The induction of cell death by EMFs and HT was further analyzed. We focused our experiments on combining EMFs and HT because of their superior effect on cancer cell viability. As shown in Table 1, treatment with EMFs and HT increases O_2_ consumption and generation of reactive oxygen species (ROS) in the cancer cells. These effects are associated with a decrease in mitochondrial membrane potential (MMP), glutathione (mtGSH), and ATP (mtATP) [all consequences of the damage caused by the increase in ROS [23]]; and also to an increase in the cytosolic caspase 3 activity (Table 1), AIF and cytochrome C (Figure 3B) [key executioners of apoptosis [24]]. These experimental facts prove that EMFs and HT activate the molecular mechanisms of mitochondria-dependent apoptotic death. It is known that inhibition of cellular energy production, generation of ROS, imbalance of cellular Ca^2+^ homeostasis, or extracellular cell death signals are all stimuli capable of inducing either apoptosis or necrosis. The relative rate of these two processes (protease and endonuclease activation versus bioenergetic catastrophe) determines whether a cell will undergo primary necrosis or apoptosis [24], a fact that usually resembles the heterogeneity of a growing cancer cell population. Despite this, EMFs and HT mainly cause cancer cell death by apoptosis, whereas only a small % corresponds to necrosis (Figure 2B). From here, it remains to be elucidated whether the activation of cell death is a direct consequence of the action of NIR on mitochondria or secondary to other mechanism(s).

### 3.3. EMFs and HT Increase Lysosomal Permeability

**Figure 4:**
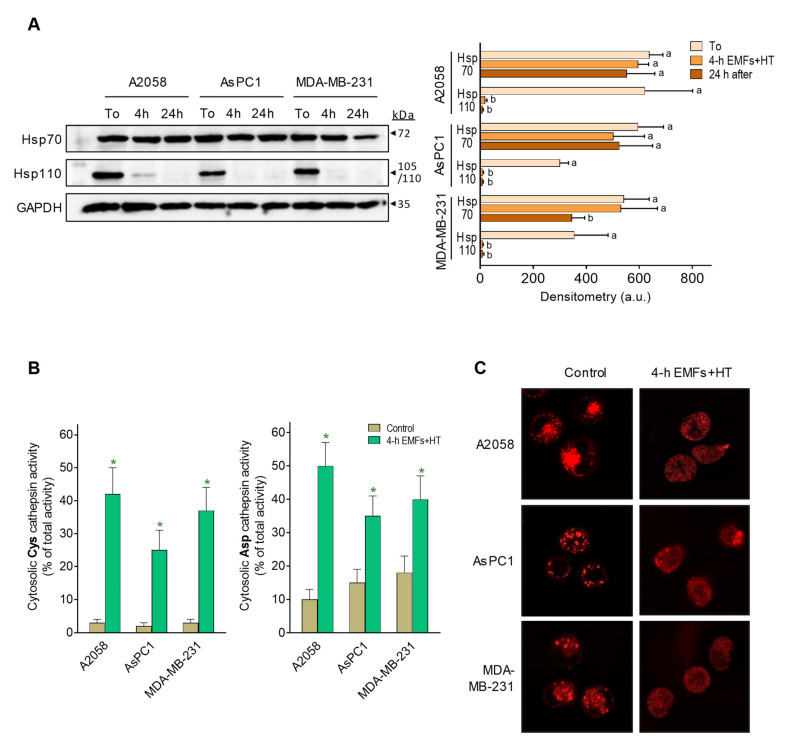
Effect of EMFs and HT on HSP70 and HSP110 and lysosomal permeability. (**A**) Protein levels (western blots) of Hsp70 and Hsp110 were measured in cancer cells after exposure to EMFs and HT (4 h protocol as in Figure 1C) and 24 h after exposure. Densitometric analysis (a.u. arbitrary units) represents the mean values ± SD for four different experiments per cell line and time point. A one-way analysis of variance (ANOVA) was used to make comparisons among the different experimental times. Different letters indicate statistical differences *p* < 0.05. (**B**) Cysteine and aspartate cathepsin activities in the cytosolic fraction were measured after exposure to EMFs and HT (4 h protocol as in Figure 1C) (*n* = 5 * *p* < 0.01 comparing EMFs and HT-treated cells versus untreated controls). (**C**) Lysosome staining (LysoTracker) was performed in the cancer cells (representative images) after the 4 h protocol (as in Figure 1C), showing EMFs and HT-induced diffusion of the lysosomal marker into the cytosol. The original western blot figures could be found in Figure S3.

The cellular heat shock (HS) response involves the heat-shock proteins (HSP). The Hsp70 is the main HSP system, provides thermotolerance, and has a central role in translation, post-translation, prevention of aggregates, and refolding of aggregated proteins [25]. Hsp110, a cofactor of Hsp70, can provide further tolerance upon cell exposure to extreme temperatures [26]. Hsp70 could be key in cancer cells since it is overexpressed in different cancers and also plays an anti-apoptotic role in favoring cancer cell survival (e.g., [27]). As shown in Figure 4A, Hsp70 levels are not significantly affected upon exposure to EMFs and HT (4 h protocol, as in Figure 1C), and only 24 h after exposure, we observed in MDA-MB-231 cells a decrease of approximately 36% as compared to controls. However, Hsp110 practically disappears after exposure to EMFs and HT, and its levels do not recover in the following 24 h period (Figure 4A). These results show, in different cancer cells, that Hsp70 levels remain at (or close to) control values despite exposure to EMFs and HT.

Nylandsted et al. [28] reported that Hsp70 is found in the lysosomes of cancer cells but rarely in those of normal cells, thus facilitating cancer cell survival by keeping lysosomal integrity. HSPs normally bind to lipid membranes and facilitate plasma membrane stabilization during stress conditions [29]. Lysosomal membrane permeabilization (LMP) associates with the release to the cytosol of cysteine and aspartate cathepsins [30], which are known inducers of apoptotic cell death [31].

As shown in Figure 4B, treatment with EMFs and HT (4 h protocol, as in Figure 1C) causes an increase in cytosolic cathepsin activities (see also Figure 4C showing lysosomal content diffusing into the cytosol), thus suggesting that this increase could be the underlying mechanism activating the mitochondria-dependent apoptotic cells death (Figure 2B). To prove this hypothesis, we silenced Hsp70 expression in AsPC1 cells before subjecting them to the effect of EMFs and HT. We used these cells as a proof of concept because of their relative resistance to EMFs + HT in the short-term (4 h) (Figure 1C). EMFs and HT (4 h protocol, as in Figure 1C) caused a decrease in wild-type AsPC1 cell viability to approximately 47% of control values (necrotic cells, based on microscopic analysis, were <4% of non-viable cells). The same EMFS and HT-induced stress in Hsp70-knockout (shRNA-dependent) AsPC1 cells drastically decreased viability to approximately 5% of control values (% of apoptotic and necrotic cells, based on microscopic analysis, was of approximately 27 and 73% of non-viable cells, respectively) (*p* < 0.01 comparing Hsp70-knockout versus wild-type AsPC1 cells, *n* = 5). The Supplementary Materials Figure S1 shows that, in AsPC1 cells treated with EMFs and HT, the activity of cytosolic cathepsins further increases in the Hsp70-knockout cell subset. The higher % of necrotic cells in Hsp70-knockout AsPC1 cells is unsurprising since massive LMP typically results in subapoptotic or necrotic cell death [30]. These data suggest a direct relationship between EMFs and HT-induced LMP, Hsp70, cathepsins, and the activation of mitochondria-dependent cell death.

### 3.4. Strategies to Complement the Anticancer Effect of EMFs and HT and Facilitate the Complete Elimination of Cancer Cells

**Figure 5:**
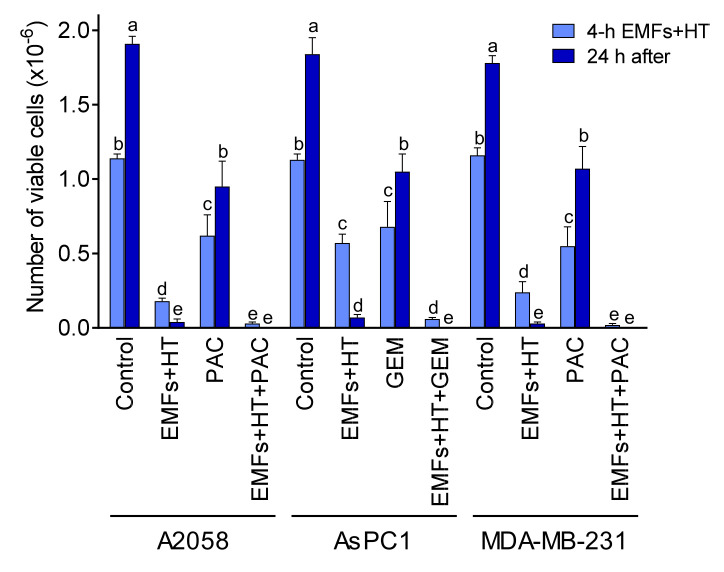
Effect of EMFs HT and chemotherapy on cancer cell viability. Cancer cells were seeded 24 h before starting the treatments. Cells were treated with EMFs and HT (4 h), as in Figure 1C. Paclitaxel (PAC 1 μM) was present in the cultured medium during the 4 h protocol (Figure 1C). Gemcitabine (GEM 25 μM) was present in the cultured medium during the last 30 min of the 4 h protocol. At the end of the 4 h treatment period, the culture medium was changed, and the cells were kept in culture for 24 additional hours. A two-way analysis of variance (ANOVA) was used to make comparisons among the different groups after 4 h of treatment with EMFs + HT and 24 h after. Different letters indicate statistical differences *p* < 0.05 (*n* = 5).

The main goal of any anticancer strategy is to eliminate all growing cancer cells completely. As shown in Figure 1C, exposure to EMFs and HT does not kill all cancer cells in our experimental conditions. In this, the marked resistance of malignant cells to a temperature as high as 52 °C is striking. Nevertheless, as explained above, the extremely low cell viability found 24 h after EMFs and HT treatment may well be misleading. Thus, in order to make our strategy as efficacious as possible, we first investigated the combination of EMFs and HT with standard chemotherapy currently in use against the types of cancers assayed [32,33,34]. As shown in Figure 5, the combination of EMFs, HT (4 h protocol as in Figure 1C), and paclitaxel (PAC, in A2058 and MDA-MB-231 cells) or gemcitabine (GEM, in AsPC1 cells) drastically decreased cancer cell viability to approximately 2.6 (A2058), 5.3 (AsPC1), and 1.7 (MDA-MB-231) % of control values. Cell viability was remeasured 24 h after exposure to the combination of EMFs + HT + chemotherapy, and no viable cell could be found (Figure 5). In this last 24 h period, the culture medium was renewed to eliminate the presence of PAC or GEM. Both drugs were incubated at concentrations (1 µM PAC, 25 µM GEM) that reflect bioavailable concentrations measured after their in vivo administration and during the period used in our in vitro assays.

PAC is a taxane that binds to tubulin, the protein component of microtubules, simultaneously promoting their assembly and disassembly to form stable, nonfunctional microtubules. It is chemotherapy used against different types of cancer, including melanoma [35]. PAC is administered at doses of 100–250 mg/m^2^ IV (24 h infusion) (http://www.cancer.org (accessed on 5 April 2022)). Taking the lowest dose of 100 mg/m^2^, and approx. 1.8 m^2^/70 kg in humans, that dose means approximately 2.6 mg/kg × 24 h or 0.107 mg/kg × h (0.428 mg/kg × 4 h). The water content in the human body is approximately 0.7 L/kg of body weight. Therefore, a patient will receive approximately 0.612 mg of PAC/L of body water × 4 h. Since 854 µg of PAC/L are equivalent to 1 µM (just slightly above the concentration expected using the dose of 100 mg/m^2^, we used 1 µM PAC in our experiments. In fact, plasma levels of unbound PAC are close to 1 µM during a period of 4–5 h after an IV dose of 135 mg/m^2^ [36].

GEM is a nucleoside analog that works by blocking the synthesis of new DNA, which results in cell death. It is a chemotherapy used to treat various carcinomas and as a first-line treatment alone for pancreatic cancer [37]. GEM is usually administered at 1000 mg/m^2^ IV infusions over 30 min once weekly × 7 weeks (http://www.cancer.org (accessed on 5 April 2022)). That means 25.7 mg/kg × 30 min or 36.7 mg of GEM/L of body water × 30 min. Although 263 µg of GEM/L is equivalent to approximately 139 µM, the mean GEM plasma concentrations range around 32 µM 30 min after a dose of 1000 mg/m^2^, 8 µM at 60 min, and undetectable levels at 120 min [38]. Based on this pharmacokinetics, we calculated a concentration of 25 µM to be added to the culture medium 30 min before finishing the 4 h period (as in Figure 1C).

**Figure 6:**
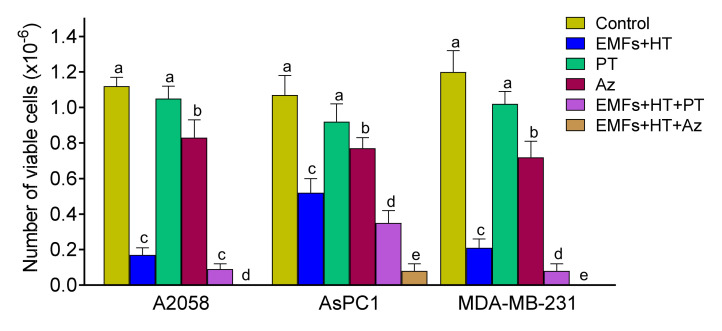
Effect of EMFs HT and a natural lysosomal membrane permeabilizer or a targeted anti-Hsp70 therapy on cancer cells viability. Cells were treated with EMFs and HT, as in Figure 1C. Pterostilbene (PT 20 μM) or apoptozole (Az 4–5 μM depending on the IC50 values described in Section 3) were added to the cultured medium right before (PT) or 12 h before starting the 4 h protocol (Az) (as in Figure 1C). A one-way analysis of variance (ANOVA) was used to make comparisons among the different experimental groups. Different letters indicate statistical differences *p* < 0.05 (*n* = 5).

Based on the EMFs and HT-induced LMP effect (see above), alternatively, we also investigated if molecules capable of increasing the LMP could improve the anticancer effect of EMFs and HT. To this aim, we assayed first a natural polyphenol, pterostilbene (PT), which has demonstrated LMP properties as well as other anticancer effects [39]. As shown in Figure 6, PT (20 µM × 4 h) alone very slightly decreased the number of viable cancer cells as compared to control values. The concentration of this polyphenol was selected based on pharmacokinetic criteria [40]. PT can be administered in vivo in the form of the disodium salt of PT phosphate (LGC Standards, Middlesex, UK) to ensure that the selected concentration of PT can reach the tumor without systemic side effects during a period equivalent to that used under in vitro conditions (Estrela JM, unpublished observations). When EMFs and HT (as in Figure 1C) were combined with PT, the number of viable cancer cells decreased to approximately 7.5 (A2058), 32.7 (AsPC1), and 6.7 (MDA-MB-231) % of control values (Figure 6).

In addition, we assayed a targeted anti-Hsp70 therapy using apoptozole (Az, N-(4-carboxamidobenzyl)-2-(3,5-bis-trifluoromethyl)-4,5-bis-(4-methoxyphenyl)-imidazole, Merck). Az is a small molecule that inhibits the ATPase activity of Hsp70 by binding to its ATPase domain without affecting other Hsp, and induces apoptotic cancer cell death via caspase activation [41]. The IC50 values of Az were 4.5 ± 0.3, 5.0 ± 0.5, and 4.0 ± 0.2 µM for A2058, AsPC1, and MDA-MB-231 cells, respectively (*n* = 5 in all cases). As shown in Figure 6, Az alone decreased the number of viable cancer cells to approximately 74 (A2058), 72 (AsPC1), and 60 (MDA-MB-231) % of control values (Figure 6).

When EMFs and HT (as in Figure 1C) were combined with Az, the number of viable cancer cells decreased to 0 (A2058), 7.5 (AsPC1), and 0 (MDA-MB-231) % of control values (Figure 6).

### 3.5. The Combination of EMFs, HT, Standard Chemotherapy, and Pterostilbene Induces a Complete Regression of Human Pancreatic Cancer Xenografts

**Figure 7:**
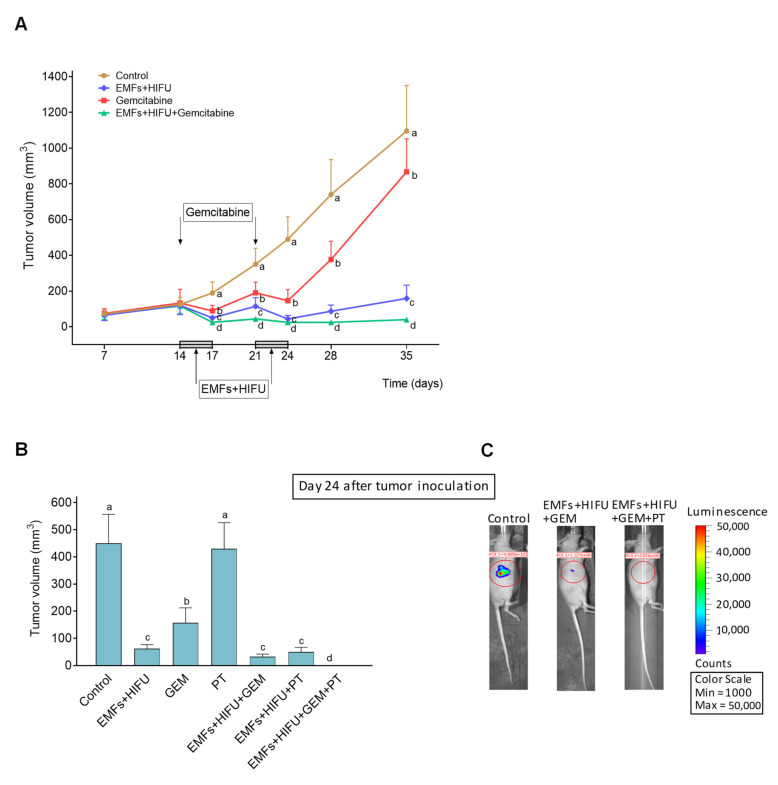
Effect of EMFs + HIFU-induced HT, gemcitabine and/or PT on the growth of AsPC1 pancreas carcinoma. Cancer cells were inoculated subcutaneously on day 0, and mice were treated with EMFs and HIFU as described under Materials and Methods. (**A**) EMFs and HIFU were applied once per day per three consecutive days (Monday to Wednesday) for two consecutive weeks starting on day 14 after tumor inoculation. Gemcitabine (50 mg/kg) was administered twice on days 14 and 21. A one-way analysis of variance (ANOVA) was used to make comparisons among the different experimental groups at each time point. Different letters indicate statistical differences *p* < 0.05 (*n* = 15 mice per experimental group). (**B**) A disodium salt of PT phosphate (Chromadex Inc. Los Angeles CA) (100 mg of PT/kg) was administered i.p. (one dose 30 min before starting each irradiation session with EMFs and HT). A one-way analysis of variance (ANOVA) was used to make comparisons among the different experimental groups. Different letters indicate statistical differences *p* < 0.05 (*n* = 12 mice per experimental group). (**C**) Representative images of mice inoculated with AsPC1/Luciferase Stable Cells and treated with EMFs HT and gemcitabine (GEM) or EMFs HT gemcitabine and PT.

As a proof of concept of our therapeutic strategy, we investigated if the combination of EMFs and HIFU-induced HT could improve the effectiveness of standard chemotherapy on a human pancreas carcinoma growing in mice. AsPC1 xenografts were treated with gemcitabine, EMFs, and HIFU-induced HT, or the triple combination. As shown in Figure 7, gemcitabine, administered at an MTD [42], slightly affected cancer growth (approximately 22% inhibition compared to controls 35 days after inoculation). The effect of treatment with EMFs and HIFU decreased cancer volume to approximately 14% of controls, whereas the combination of EMFs + HIFU + gemcitabine decreased cancer volume to approximately 3–4% of controls 35 days after inoculation. Importantly, in our experimental conditions, no additional side effects were observed when EMFs and HT were added to the chemotherapy. These results demonstrate the potential efficacy of our strategy. The drastic reduction of pancreatic cancer growth (Figure 7A) may facilitate its elimination by surgery or by treating the tumor-bearing animal/patient with an additional targeted therapy (as suggested above). Based on the data reported in Figure 6, we added PT to the combination of EMFs + HT + gemcitabine. A disodium salt of PT phosphate was administered i.p. (as indicated in the caption of Figure 7). The concentration of PT in the growing tumor was 118 ± 27 µM 30 min after its administration, 59 ± 12 µM (*n* = 7) at 60 min, and 14 ± 4 µM (*n* = 7) at 120 min (*n* = 7 in all cases). As shown in Figure 7B, complete tumor regression was achieved using the combination of EMFs + HT + gemcitabine + PT, and no tumor cell activity was detected using AsPC1 cells transfected with luciferase (Figure 7C). Mice treated with EMFs + HT + gemcitabine + PT (Figure 7C) were followed up, but tumor growth activity (assayed once a week with the luciferine-luciferase assay) did not recover even 2 months after the completion of the treatment. Two months after treatment, all mice followed health evaluation based on the NIH standard methodology (i.e., animal weight and complete blood cell count and chemistry) (www.nih.gov (accessed on 14 October 2022)). Results were similar in control untreated mice and tumor-bearing mice 2 months after the treatment to eradicate the growing cancer (Supplementary Materials Table S1). Moreover, data comparing untreated tumor-bearing mice and treated tumor-bearing mice (at day 25, one day after treatment, see Table S1 and Figure 7A) were not significantly different. This means that changes in weight, hematology, and clinical chemistry (Table S1) are associated with the growth of the tumor and are not a consequence of the treatment. Our data indicate that the therapy is safe and does not compromise key parameters linked to the health status of mammals.

## 4. Discussion

Do EMFs cause heating of the cells? The mechanism by which the oscillating magnetic field may cause heating of the tissue is by inducing Foucault (or Eddy) currents in the tissue [43]. These currents revolve around the magnetic field lines in the tissue and, by the Joule effect, could heat the tumor cells. This heating effect is related to the conductivity (σ) of the living tissue. This conductivity provides the path for microscopic Eddy currents, which flow in circular paths. The power per unit mass (P) that heats the cells is given by the following equation: P = π^2^·B^2^·d^2^·f^2^/(6·ρ·D). Where B is the magnetic flux density, d is the depth of tissue over which the magnetic field is provided, f is the field frequency, ρ is the tissue resistivity (inverse to the electrical conductivity), and D is the mass density of the tissue [44].

The conductivity of tumor tissue can be up to five times higher than the conductivity of healthy tissues and has an approximate value of 0.15 Siemens/m at 100–300 kHz [45]. D for biological tissue is variable (between 900–1050 kg/m^3^) but can be approximated by that of the water, 1000 kg/m^3^ [46]. In our experimental setup, the value for *p* is <20 pW/Kg, which is very low. Therefore, the mechanism related to the EMF’s effect is not due to heating. The synergic effect with HT could be attributable, at least in part, to an increase in the conductivity associated with an increase in the mobility of the charged molecules.

Methods of heating used for cancer treatment involve (a) electromagnetic heating [i.e., in ascending order of frequency and descending penetration depth, capacitive (using metal electrodes and 8–25 MHz) or radiative radiofrequency (using extracorporeally placed antennas with operating frequencies ranging from 60 MHz to 150 MHz), microwave heating (400–2500 MHz), and infrared (using infrared lamps, frequency > 300 GHz) and laser heating]; (b) ultrasounds (acoustic energy at frequencies 0.5–10 MHz); (c) hyperthermic perfusion, generally combined with chemotherapy; (d) conductive heating, as interstitial implants of metal needles with hot water and palladium–nickel thermoseeds; and (e) magnetic nanoparticles, exposed to an external magnetic field (0.1–0.2 MHz) [7]. All these methods have pros and cons. HIFU refers to intensities >5 W/cm^2^, which produce thermal and mechanical effects, generating a localized temperature increase in the tissues. HIFU administration allows a precise treatment of targeted areas, where injury to the surrounding tissue will depend on the temperature reached and the time of exposure. In this regard, since the ultrasonic energy is focused on a specific volume of tissue, it is key to consider that the temperature will decrease (based on a Gaussian model) as we move away from that specific volume. With the purpose of making an approximation of the effects of the two NIR on a normal cell, we applied our protocol to 3T3 fibroblasts (www.atcc.org (accessed on 5 July 2021)). As shown in Figure S2A,B, EMFs (100 kHz, as in Figure 1C) or HT at 42 °C (40 min, as in Figure 1C) did not significantly affect 3T3 viability. HT at 47 °C or 52 °C decreased 3T3 viability to approximately 69% and 29%, respectively, of control values (Figure S2B). As shown in Supplementary Figure S2C, EMFs (100 kHz) and HT at 42 °C did not decrease 3T3 viability compared to control values. A combination of EMFs (100 kHz) and HT at 47 °C decreased 3T3 viability to approximately 54% of control values, whereas EMFs (100 kHz) and HT at 52 °C further decreased 3T3 viability to approximately 15% of control values (4 h protocol, Figure S2C). Nevertheless, a gradation in the level of damage dependent on temperature is evident. Hence, it is key to focus the energy that generates HT in the tumor and use several energy beams, thus preserving as much normal tissue as possible. In addition, as shown in Figure S2D, EMFs + HT-induced cell death in 3T3 fibroblasts is mainly apoptotic (as in the cancer cells, see Figure 2B and Figure 3B). In unstressed 3T3 fibroblasts, Hsp70 and Hsp110 levels are lower than in the cancer cells, whereas, as a consequence of their exposure to EMFs and HT (47–52 °C), both Hsp70 and Hsp110 practically disappear (Figure S2E,F). In the case of Hsp70, this is different from what we observed in cancer cells (Figure 4A), suggesting the need for further studies comparing cancer cells and their normal counterparts when subjected to the combination of the two non-ionizing energies.

In practice, the combination of EMFs and HIFU may offer the following advantages: (a) RF EMFs have not been reported as having any significant toxicity for normal tissues; (b) magnetic resonance guided-HIFU (MRgFUS) can target specific volumes of cancers (as small as a few mm of diameter); (c) HIFU can be applied using a fast array program (once the target is localized, and the volume and shape of the tumor reconstructed in a computer system, the program can design the sequential application of HIFU at a series of specific points, thus maximizing efficiency; (d) to work with EMFs and HIFU allows to limit the rise in temperature to a level that may better preserve the surrounding normal tissues; (e) although the number of transducers may need to be increased, depending on the location of the tumor to be treated, a holographic design of the HIFU piezoelectric transducer units (e.g., patent WO2020/084181A1) can reduce their number and, thus, simplify the system; (f) the advantage of using multiple transducers is that the energy emitted by each one cannot damage normal tissues, but it can be concentrated in the growing cancer; (g) if needed, EMFs and HIFU could be applied several times (e.g., once per day) in order to maximize their efficacy in vivo. However, in determining the number of energy beams needed to avoid an excessive rise in temperature in normal tissues (while heating up the cancer to 52 °C), some important issues will have to be considered, i.e., (a) computational models or simulations that take into account the organ’s geometry, specific location, tissue properties, and energy propagation characteristics; (b) overlapping beams can cause cumulative heating effects and potentially raise the temperature beyond the desired limit; (c) the power and duration of each individual beam must be adjusted to control the amount of energy delivered to the target and minimize heating in the normal tissues; (d) real-time monitoring of temperature during the procedure is essential to ensure accurate control; (e) different tissues have varying thermal conductivities, which impact how heat spreads through them; (f) the specific absorption rate, or amount of energy absorbed per unit mass of tissue, and the heat capacity of each organ and tissue. In our procedure, the spatial shape of the HIFU beam was Gaussian, providing enough accuracy in heating the tumor. Despite this, we must expect HT-induced damage to the nearest peritumoral tissue. Damage comparable to the resection of non-cancerous peritumoral tissue that, as a safety margin, is carried out by surgeons during a resection. Based on the targeting accuracy of ultrasound imaging-guided robotic HIFU systems for the treatment of solid tumors [47] and the physiological mechanisms of heat loss in tissues, the temperature a few mm (<5 mm) off target will drop drastically. Therefore, the combination of EMFs and HIFU can be implemented to transfer our findings to working in vivo applications (see Figure 7). Moreover, both EMFs and HIFU are non-invasive techniques that can be further combined with other anticancer therapies (see under Results). In this regard, the present contribution offers some effective options. EMFs and HT can be combined with conventional chemotherapy (Figure 5 and Figure 7). On the other hand, EMFs and HT can also be combined with LMP inducers, such as PT or a specific anti-Hsp70-targeted drug (Figure 6). At present, PT has been assayed in clinical trials for different indications, but it has never been administered intravenously to humans. Interestingly, oral administration of PT cocrystals (as those of PT and picolinic acid, http://www.circescientific.com (accessed on 14 March 2022)), which increases PT bioavailability up to 5–10 times compared to that of the natural stilbene alone, can avoid the need to use the intravenous administration. Moreover, achieving the intratumoral concentration of the polyphenol is necessary to increase lysosomal permeability. In favor of the therapeutic use of PT is its potential to decrease Nrf2-dependent antioxidant defenses in cancer cells [22]. On the other hand, trials involving an anti-Hsp70 targeted therapy are still in their early beginning. Furthermore, recently, some anticancer lysosomotropic drugs (e.g., nortriptyline, siramesine, and desipramine) and their nanoformulations have been engineered to accumulate specifically within these organelles. These drugs can enhance LMP or disrupt the activity of resident enzymes and protein complexes, such as v-ATPase and mTORC1 [48] (a list of inducers of lysosomal cell death can be found at, e.g., [49]). Mechanistically, an increase in cytosolic cathepsin activity triggers the mitochondrial membrane permeabilization through cleavage of Bid or via activation of phospholipase A2 and the consequent increase in araquidonic acid [50]. Furthermore, cathepsins can directly cause chromatin condensation [51], whereas the cytosol acidification can lead to L-DNAase II activation and chromatinolysis [52].

Another interesting aspect that deserves further investigation is the possible role of mitochondrial HSP in these mechanisms. ROS generation increases with temperature [53] (Table 1). Thus, it is possible that mitochondrial HSP (Hsp70 in particular), in addition to their roles in protein transport and folding, protects mitochondrial proteins and DNA from thermal and ROS damage.

Initially, no reasons may preclude multiple combinations, e.g., EMFs + HT + chemotherapy + a lysosomal permeabilizer, which is also a feasible option in case of finding an unexpected cancer cell resistance in vivo. In all this, preclinical studies and clinical trials will be necessary steps. Naturally, there are no strict restrictions in order to combine EMFs and HT with other available (or still being implemented) cancer-type-specific therapeutic options, e.g., immunotherapy or signaling-related targeted therapy. It is also important to bear in mind the potential counterproductive effects of some lysosomal stabilizers, such as acetylsalicylic acid or hydrocortisone [54]. These types of drugs should be avoided during cancer treatment with our strategy.

Despite differences in genetic backgrounds and in vivo behavior among cancer cells, the combination of EMFS and HT seems to affect them in a similar way (see, e.g., Figure 1C) and based on the same mechanism (see, e.g., Table 1 and Figure 4). Nevertheless, Hsp70 levels are a clear example of a mechanism of resistance to HT. Hsp70 inhibits the mitochondrial outer membrane permeabilization, thus reducing caspase activation and neutralizing the AIF [55]. Moreover, Hsp70 also localizes to lysosomal membranes and can protect LMP induced by different stimuli [56]. Therefore, although eventual mechanisms of resistance to EMFs and HT should be explored in depth, it is encouraging the fact that both energies, if applied at the correct conditions and time, appear highly effective both in vitro and, as shown in Figure 7, in vivo.

## 5. Conclusions

This work demonstrates that a combination of EMFs and HT causes irreversible damage to different cancer cells (i.e., melanoma, pancreatic cancer, and breast cancer). The mechanism involves permeabilization of the lysosomes, the release of cathepsins to the cytosol, and activation of the mitochondria-dependent cell death. A combination of EMFs and HT with standard chemotherapy, molecules that further promote lysosomal permeabilization, and/or targeted anti-Hsp70 therapy can completely kill cancer cells. This strategy, supported by in vitro and in vivo evidence, may complement current oncotherapies and can be applied to different cancers. In vivo treated mice followed post-treatment health evaluation (NIH standard methodology), which showed that in our experimental conditions, the therapy is safe and, per se, does not compromise mouse physiology.

## Figures and Tables

**Table 1:** Effect of EMFs and HT on ROS generation and the molecular mechanisms of apoptosis.

	A2058		AsPC1		MDA-MB-231	
	-	+ EMFs + HT	-	+ EMFs + HT	-	+ EMFs + HT
O_2_ consumption (pmol/10^6^ cells × min)	627 ± 79	1067 ± 165 **	784 ± 102	1226 ± 188 **	551 ± 82	1046 ± 124 **
H_2_O_2_ (nmol/10^6^ cells × min)	0.77 ± 0.10	1.56 ± 0.31 **	0.94 ± 0.15	1.47 ± 0.27 *	0.62 ± 0.13	1.24 ± 0.29 **
O_2_^−^ (nmol/10^6^ cells × min)	0.33 ± 0.04	0.69 ± 0.12 **	0.45 ± 0.07	0.72 ± 0.15 **	0.26 ± 0.05	0.48 ± 0.09 **
MMP (TPM accumulation ratio, %)	100 ± 5	42 ± 15 **	100 ± 4	56 ± 16 **	100 ± 6	35 ± 12 **
mtGSH (nmol/10^6^ cells)	4.2 ± 0.9	2.0 ± 0.5 **	2.8 ± 0.6	1.3 ± 0.5 **	3.5 ± 0.7	1.5 ± 0.5 **
mtATP (mM)	1.05 ± 0.10	0.52 ± 0.14 **	0.96 ± 0.12	0.41 ± 0.09 **	0.92 ± 0.13	0.33 ± 0.08 **
Caspase 3 (pmol/10^6^ cells x min)	1.87 ± 0.46	3.66 ± 0.39 **	1.67 ± 0.35	3.15 ± 0.42 **	2.05 ± 0.51	4.14 ± 0.67 **

All parameters were measured in cancer cells after exposure to EMFs and HT (4 h protocol as in Figure 1C). * *p* < 0.05, ** *p* < 0.01 comparing EMFs + HT versus untreated controls (*n* = 5–6).

## Data Availability

All raw data regarding these studies are available in the Figshare repository (https://doi.org/10.6084/m9.figshare.22220065 (accessed on 6 March 2023)).

## Supplementary Materials

The following supporting information can be downloaded at https://www.mdpi.com/article/10.3390/cancers15133413/s1, Figure S1: Effect of shRNA-induced downregulation of Hsp70 on the EMFs and HT-induced increase in cytosolic cathepsin activities in AsPC1 cells; Figure S2: Effect of EMFs and HT on 3T3 fibroblasts; Figure S3: Whole blots: (A) Figure 3C, (B) Figure 4A, (C) Figure S1, (D) Figure S2E; Table S1: Hematology and clinical chemistry data in AsPC1-bearing mice treated to induced suppression of the growing tumor.
