# Supplementary material for: Externally Applied Electromagnetic Fields and Hyperthermia Irreversibly Damage Cancer Cells
Source: Cancers (Basel). 2023 Jun 29;15(13):3413. doi: 10.3390/cancers15133413 (PMC10340829; doi:10.3390/cancers15133413)
Supplement: Supplementary file 1 [file cancers-15-03413-s001.zip › cancers-2410337-supplementary.pdf]

Supplementary Materials

Externally Applied Electromagnetic Fields and Hyperthermia Irreversibly Damage Cancer Cells

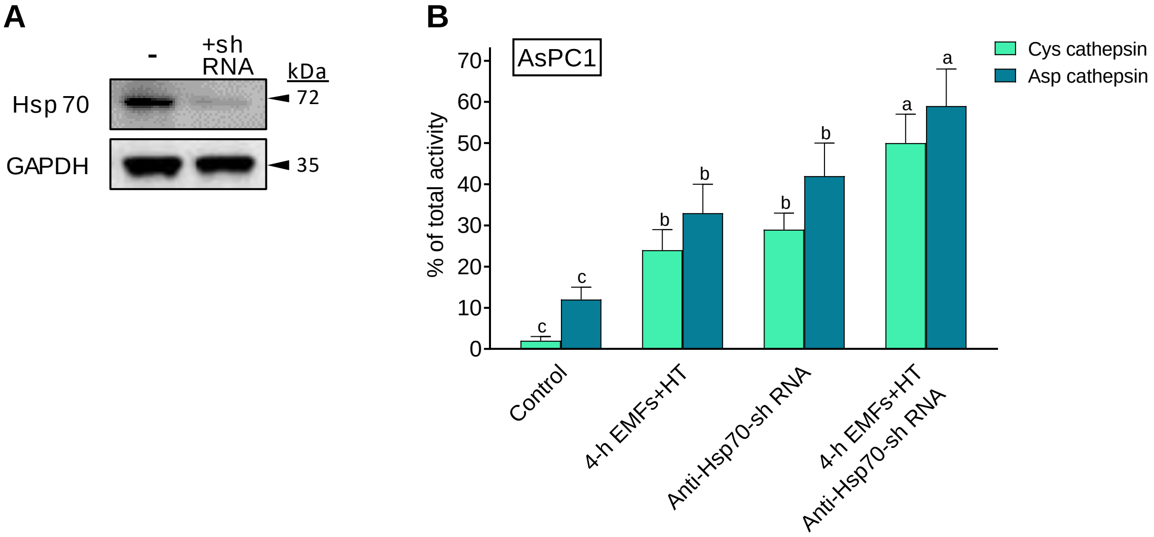

Figure S1. Effect of shRNA-induced downregulation of Hsp70 on the EMFs and HT-induced increase in cytosolic cathepsin activities in AsPC1 cells. (**A**) Hsp70 protein levels (western blots) were measured in control and EMFs and HT-treated AsPC1cells pretreated with anti-Hsp70-shRNA (n = 4 $p < 0.01$ comparing EMFs and HT-treated cells versus untreated controls). (**B**) Cathepsin activities in the cytosolic fraction were measured after exposure to EMFs and HT (4h-protocol as in Figure 1C). A one-way analysis of variance (ANOVA) was used to make comparisons among the different experimental conditions for each cathepsin activity. Different letters indicate statistical differences $p < 0.05$. (n = 4-5).

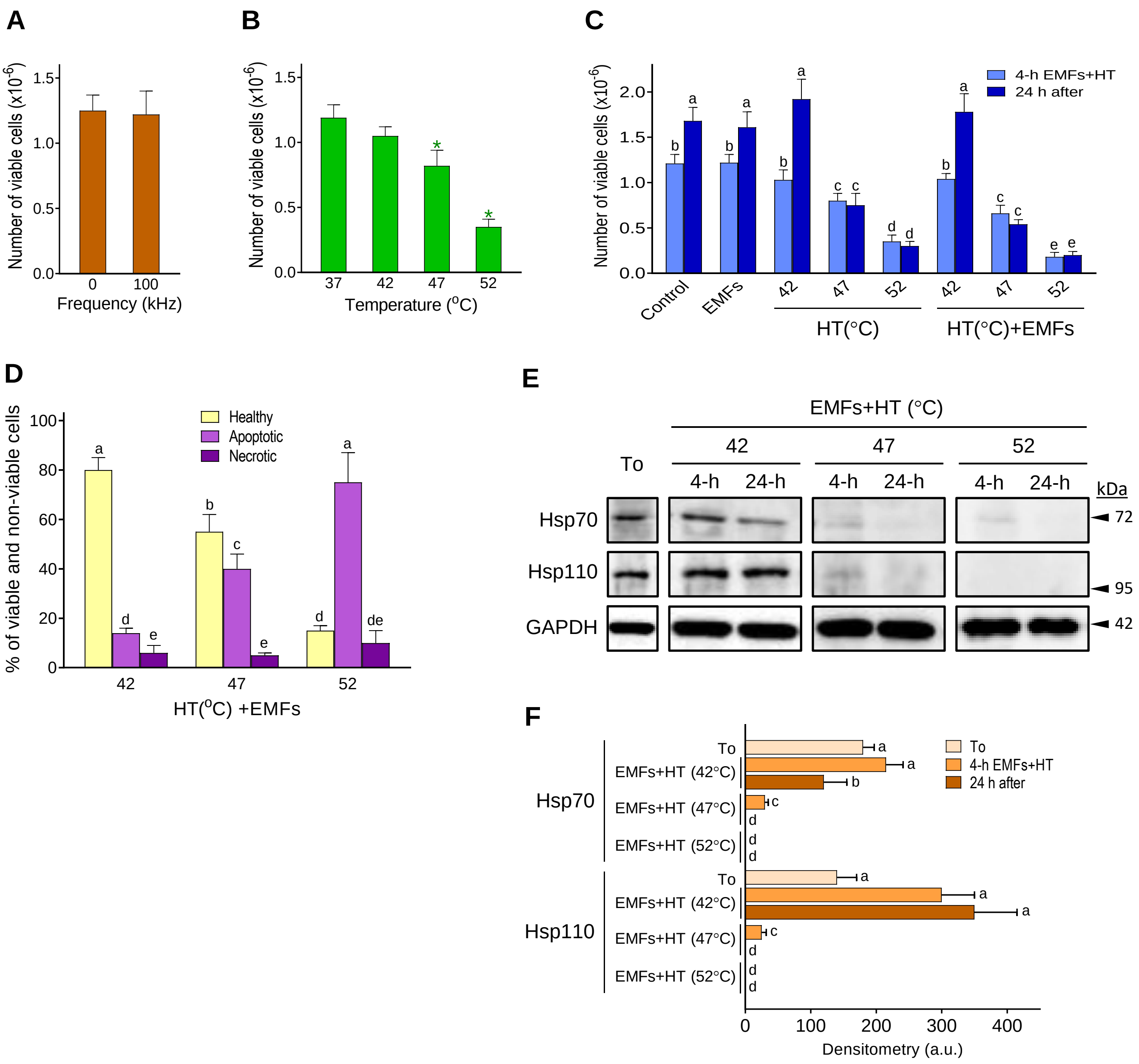

Figure S2. Effect of EMFs and HT on 3T3 fibroblasts. (**A**) Effect of EMFs. 3T3 cells were seeded and 24 h later exposed to EMFs (100 kHz x 4 h, as in Figure 1C) (n = 4). (**B**) Effect of HT. 3T3 cells were seeded (20,000 cells/cm²) and 24 h later exposed to HT (42-52°C x 40 min). * $p < 0.01$ comparing all conditions versus controls (37°C) (n = 4 t test). (**C**) Effect of EMFS and HT. Cancer cells were seeded and 24 h later exposed to EMFs (100 kHz x 4h) and HT (42-52°C x 40 min from min 120 to min 160 of the 4h-period where cells were constantly exposed to the EMFs). Then surviving cells were cultured for 24 additional hours without further exposure to EMFs and HT. A two-ways analysis of variance (ANOVA) was used to make comparisons among the different groups after 4 h of treatment with EMFs+HT and 24 h after. Different letters indicate differences $p < 0.05$ (n = 4). (**D**) Cell death analysis after the 4-h protocol (Figure 1C) based on Hoechst 33342 and propidium iodide (see under Methods). Different letters indicate statistical differences p < 0.05 (n = 4). (**E**) Effect of EMFs and HT on HSP70 and HSP110. Protein levels (western blots) of Hsp70 and Hsp110 were measured in 3T3 cells after exposure to EMFs and HT (4-h protocol as in Figure 1C) and 24h after exposure. (**F**) Densitometric analysis (a.u. arbitrary units) represents the mean values ± SD for 4 different experiments per time point. A one-way analysis of variance (ANOVA) was used to make comparisons among the different experimental times. Different letters indicate statistical differences $p < 0.05$.

Table S1. Hematology and clinical chemistry data in AsPC1-bearing mice treated to induced suppression of the growing tumor. Full treatment means the combination of EMFs+HIFU+GEM+PT as in Figure 7B. A one-way analysis of variance (ANOVA) was used to make comparisons among the different experimental groups. Different letters indicate statistical differences $p < 0.05$ (n = 7 mice per experimental group).

	**Non-tumor bearing mice**	**Tumor-bearing mice**	
		**One day after treatment**	**Two months after treatment**
Animal weight (g)	25.0 ± 1.2^b	20.5 ± 0.9^c	29.3 ± 1.5^a
**Hematology**			
Hematocrit (%)	38.7 ± 0.6^a	25.4 ± 1.3^b	37.5 ± 0.9^a
Hemoglobin (g/dL)	13.8 ± 0.2^a	8.6 ± 0.5^b	13.5 ± 0.4^a
Erythrocytes ($10^6/\mu L$)	8.9 ± 0.3^a	5.3 ± 0.4^b	8.4 ± 0.5^a
Mean red cell volume (μm^3)	41.7 ± 0.5^a	40.6 ± 0.6^b	42.1 ± 0.5^a
Mean red cell hemoglobin (pg)	15.0 ± 0.3^a	14.5 ± 0.4^a	15.1 ± 0.3^a
Platelets ($10^3/\mu L$)	496 ± 27^a	184 ± 45^b	480 ± 33^a
Leukocytes ($10^3/\mu L$)	7.0 ± 1.0^a	2.1 ± 0.5^b	6.5 ± 0.8^a
Neutrophiles ($10^3/\mu L$)	1.3 ± 0.2^a	0.4 ± 0.1^b	1.2 ± 0.3^a
Lymphocytes ($10^3/\mu L$)	5.2 ± 0.6^a	1.3 ± 0.5^b	5.1 ± 0.4^a
Monocytes ($10^3/\mu L$)	0.2 ± 0.05^a	0.1 ± 0.05^a	0.2 ± 0.1^a
Eosinophiles ($10^3/\mu L$)	0.1 ± 0.05^a	0.02 ± 0.01^b	0.1 ± 0.05^a
Basophiles ($10^3/\mu L$)	0.0 ± 0.0^a	0.0 ± 0.0^a	0.0 ± 0.0^a
**Clinical chemistry**			
Urea (mg/dL)	50.5 ± 3.0^b	56.7 ± 2.5^a	51.4 ± 2.2^b
Uric acid (mg/dL)	2.7 ± 0.5^a	1.3 ± 0.2^b	2.4 ± 0.4^a
Total protein (g/dL)	5.7 ± 0.2^a	5.4 ± 0.3^a	5.6 ± 0.2^a
Albumin (g/dL)	4.5 ± 0.2^a	4.3 ± 0.1^a	4.5 ± 0.2^a
Creatinine (mg/dL)	0.6 ± 0.02^b	0.7 ± 0.03^a	0.6 ± 0.02^b
Glucose (mg/dL)	207 ± 25^a	151 ± 18^b	218 ± 26^a
Total bilirubin (mg/dL)	0.5 ± 0.2^a	0.8 ± 0.2^a	0.5 ± 0.1^a
Direct bilirubin (mg/dL)	0.1 ± 0.02^b	0.2 ± 0.03^a	0.1 ± 0.02^b
Asp aminotransferase (IU/L)	220 ± 17^b	576 ± 102^a	239 ± 30^b
Ala aminotransferase (IU/L)	9.4 ± 2.1^c	178 ± 26.4^a	36.0 ± 10.5^b

γ-Glutamyl transpeptidase (IU/L)	2.0 ± 0.5[c]	14.5 ± 3.7[a]	3.5 ± 0.6[b]
Alkaline phosphatase (IU/L)	151 ± 29[b]	484 ± 86[a]	187 ± 38[b]
Lactate dehydrogenase (IU/L)	405 ± 55[b]	1136 ± 277[a]	439 ± 61[b]
Sodium (mEq/L)	147 ± 15[a]	156 ± 19[a]	155 ± 21[a]
Potassium (mEq/L)	7.7 ± 0.7[a]	7.9 ± 0.8[a]	7.5 ± 0.7[a]
Chloride (mEq/L)	110 ± 8[a]	105 ± 7[a]	107 ± 11[a]

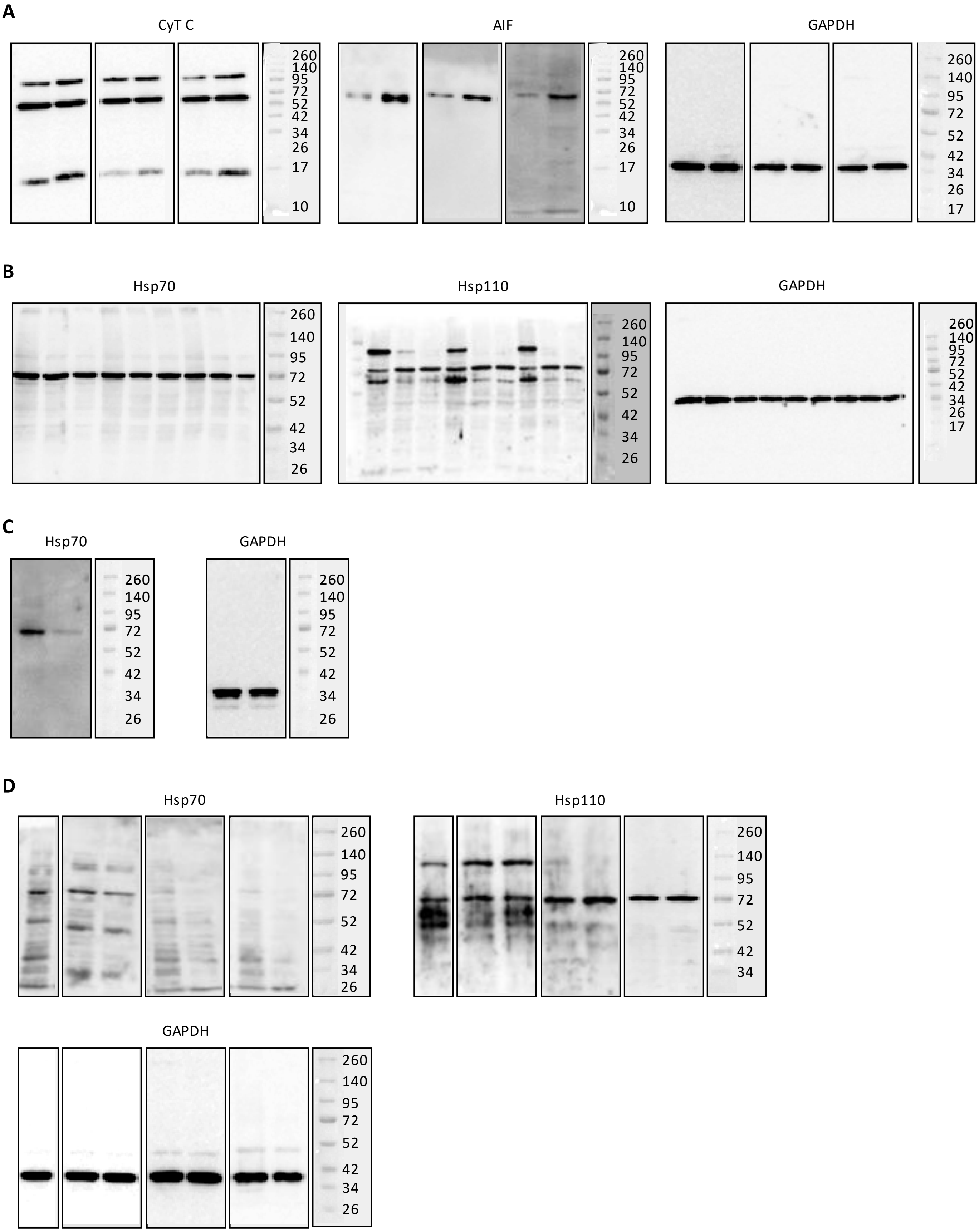

Figure S3. Whole blots: (**A**) Figure 3C, (**B**) Figure 4A, (**C**) Figure S1 and (**D**) Figure S2E.
